# Metabolic profile of women with PCOS in Brazil: a systematic review and meta-analysis

**DOI:** 10.1186/s13098-021-00636-5

**Published:** 2021-02-16

**Authors:** Poli Mara Spritzer, Ramon Bossardi Ramos, Lucas Bandeira Marchesan, Monica de Oliveira, Enrico Carmina

**Affiliations:** 1 Gynecological Endocrinology Unit, Division of Endocrinology, Hospital de Clínicas de Porto Alegre, Porto Alegre, RS Brazil; 2 Department of Physiology, Universidade Federal Do Rio Grande Do Sul, Porto Alegre, RS Brazil; 3 Post-Graduate Program in Endocrinology, Medicine School, Universidade Federal Do Rio Grande Do Sul, Porto Alegre, RS Brazil; 4 Instituto de Medicina Integral Professor Fernando Figueira Hospital, Recife, Pernambuco, Brazil; 5 University of Palermo School of Medicine, Palermo, Italy; 6 Present Address: Department of Molecular and Cellular Physiology, Albany Medical College, Albany, NY USA; 7 Division of Endocrinology, Hospital de Clínicas de Porto Alegre, Rua Ramiro Barcelos, 2350, Porto Alegre, RS 90035-003 Brazil

**Keywords:** Polycystic ovary syndrome, Obesity, Blood pressure, Insulin resistance, Metabolic abnormalities, Brazil

## Abstract

**Background:**

Polycystic ovary syndrome (PCOS) is a common endocrine disease affecting women of reproductive age and associated with reproductive and metabolic dysfunction. Few studies are available regarding metabolic traits in Brazilian women with PCOS. The aim of this systematic review and meta-analysis was to summarize the available evidence regarding metabolic traits and comorbidities in Brazilian women with polycystic ovary syndrome (PCOS).

**Methods:**

We systematically searched PubMed, Cochrane Central Register of Controlled Trials, and Embase for cross-sectional, case–control, or cohort studies focusing on populations of different regions from Brazil, published until July 31, 2019. Studies were selected if they reported PCOS diagnostic criteria. Studies without a control group were included if they presented relevant metabolic data.

**Results:**

Of 4856 studies initially identified, 27 were included in the systematic review and 12 were included in the meta-analysis, for a total of 995 women with PCOS defined by Rotterdam criteria and 2275 controls from different regions of Brazil. Obesity, metabolic syndrome and IGT were prevalent, and standard mean differences for BMI (SMD 0.67, 95% CI, 0.29, 1.05), waist circumference (SMD 0.22, 95% CI 0.02, 0.41), systolic (SMD 0.66, 95% CI 0.30, 1.01) and diastolic blood pressure (SMD 0.55, 95% CI 0.24, 0.87), glucose (SMD 0.21, 95% CI 0.04, 0.38) and HOMA (SMD 0.78, 95% CI 0.52, 1.04) were significantly higher in Brazilian women with PCOS compared to controls. Lipid profile was more adverse in PCOS vs. non-PCOS women. Between-study heterogeneities were low/moderate for glucose and HOMA and moderate/high for the other variables.

**Conclusions:**

The data of this systematic review and meta-analysis indicate that Brazilian women with PCOS have a worse metabolic profile than women without PCOS with no important regional differences. The prevalence of metabolic changes is intermediate in Brazil vs. other countries.

## Background

Polycystic ovary syndrome (PCOS) is the most common endocrine disorder among women of reproductive age [1, 2]. Despite an uncertain etiology, there is strong evidence that complex interactions between genetic, environmental, and behavioral factors contribute to the onset and to the heterogeneous expression of the syndrome [3]. In fact, not only does the syndrome involve several different phenotypes; the prevalence of the different phenotypes also varies according to ethnic groups [4–11].

Evidence from various geographic regions indicates that the metabolic features of PCOS are particularly influenced by ethnic background and behavioral characteristics. For example, obesity is particularly common in women with PCOS from the U.S [8, 12–15], but relatively uncommon in Eastern Asia [16] and in Mediterranean countries [5–7]. Impaired fasting glucose, impaired glucose tolerance, and type 2 diabetes seem particularly common in India [17, 18] and in the U.S. [13, 14] and relatively uncommon in Mediterranean countries [6, 7]. A similar pattern has been reported for the prevalence of metabolic syndrome and dyslipidemia [7, 14, 19].

In contrast, only limited information is available regarding the phenotype distribution and the metabolic expression of PCOS in some other regions, such as Brazil, a large country representing a range of ethnic backgrounds. Brazil also presents socio-economic disparities among its five regions. Total Brazilian population, according to 2020 national data, is of 211,755,692 inhabitants, with 83% distributed in the Northeast, Southeast and South (https://www.ibge.gov.br/estatisticas/sociais.html). Regarding education, inhabitants of the southeast region have the better access to education, with 28.9% of population with 25 years old or more having concluded school, while the lowest index is in the northeast region, 23.5%. Regarding economic status, the median monthly income per person in the South is U$211.3, U$187.4 in Southeast, U$184.8 in Midwest, U$94.6 in North and U$ 93.7 in Northeast. However, there is great variation in incomes according to social classes, in the whole territory.

Therefore, the aim of the present systematic review and meta-analysis was to examine the available evidence regarding the prevalence of metabolic alterations in Brazilian women with PCOS.

## Methods

### Search strategy and study selection

This systematic review and meta-analysis was registered in PROSPERO under number CRD42016038537. PubMed, Cochrane Central Register of Controlled Trials, and Embase were searched for cross-sectional, case–control, cohort, and prevalence studies published until July, 2019 and including populations from different regions of Brazil. No limits were set on publication date or language. Medical subject headings (MeSH) used in the search are presented as Additional file 1. Additional searches were performed in recent review articles and original studies with a focus on PCOS.

Studies were selected for the present review if they provided a clear definition of the criteria used for diagnosis of PCOS and analyzed of at least one of the following variables: body mass index (BMI), waist circumference (WC), blood pressure, lipid profile, glucose, HOMA-IR, metabolic syndrome (MetS), diabetes mellitus (DM), prevalence of PCOS, and milder phenotypes.

### Data extraction and quality control assessment

Two reviewers (RBR and PMS) independently screened titles/abstracts for selection of articles for full-text review. Disagreements were resolved by consensus discussion. The full text of selected articles was independently reviewed by the two authors. If selected articles were published in other languages than English or Portuguese a translate site would be used. If data were duplicated or reported more than once, the most complete study was chosen. If the required data were not located in the published article, authors were contacted to provide the missing information.

The following information was extracted from studies: name of authors, publication year, country, type of study, population characteristics, diagnostic criteria, total population, and outcomes of interest in PCOS and control group. Three authors extracted the data from each report independently. The Newcastle–Ottawa scale (NOS) was used to assess the quality of the observational studies included in the meta-analyses.

### Statistical analysis

The standardized mean difference (SMD) with 95% confidence interval (CI) was estimated using a DerSimonian and Laird (DL) random effects model. A p value of less than 0.05 was considered as statistically significant. Variables of interest were included in the meta-analysis if they were present in at least two studies.

We assessed heterogeneity from the Mantel–Haenszel model and I^2^ values (the percentage of variance in the pooled estimate due to between-study differences), with I^2^ > 50% suggesting moderate heterogeneity and p < 0.10 in Cochran’s Q test indicating significant heterogeneity[20]. The risk of publication bias was assessed using funnel plot graphics, analyzed both visually and with the Egger test. The significance of the intercept was evaluated by the t test, with p < 0.10 indicating significant publication bias [21].

Statistical analyses were performed using R version 3.4.3. (2017–11-30) (http://www.r-project.org). The metafor package for doing meta-analysis was used within the R environment. Graphs were also created using metafor[22].

## Results

### Flowchart of study selection

**Fig. 1:**
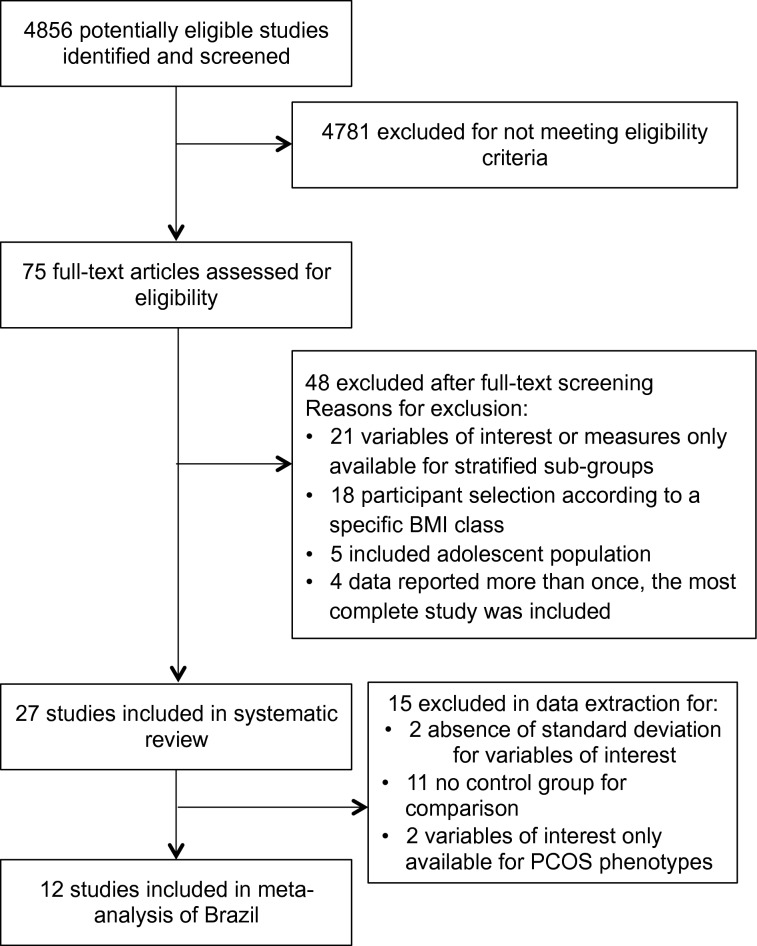
PRISMA flow diagram of the study selection process

Figure 1 provides details of the study selection. Our search yielded 4856 articles, of which 27 studies, all observational, were eligible for inclusion in the systematic review, 14 cross-sectional, 12 case–control studies and 01 cohort (Table 1). All articles were published between 2004 and 2019. Sample sizes ranged from 10 to 288 in PCOS groups and 10 to 1,500 in control groups.

**Table 1:** Characteristics of the studies included in the systematic review about Brazilian women with PCOS

Name, Year	Region	PCOS criteria	Type of studies	N (PCOS/controls)	Age (PCOS/controls
*BMI-unmatched studies*					
Avila et al. 2014 [30]	Southeast	Rotterdam	Cross-sectional	100/–	25.7 ± 4.9/–
Azevedo et al. 2011 [23]	Northeast	Rotterdam	Cross-sectional	113/242	26.2 ± 4.3/ 26.8 ± 5.0
Carvalho et al. 2017 [31]	Southeast	Rotterdam	Case–control	86/86	31.1 ± 4.92/29.0 ± 7.04
Cerqueira et al. 2010 [24]	Northeast	Rotterdam	Cross-sectional	56/54	26.2 ± 6.0/27.7 ± 6.1
Costa et al. 2012 [29]	Northeast	Rotterdam	Cross-sectional	113/–	27.2 ± 4.5/–
de Medeiros et al. 2014 [48]	Midwest	Rotterdam	Cross-sectional	288/–	26.9 ± 5.5/–
Gabrielli et al. 2012 [26]	Northeast	Rotterdam	Cross-sectional	73/725	28.4 ± 6.5/31.0 ± 7.3*
Graff et al. 2017 [44]	South	Rotterdam	Case–control	84/54	23.5 ± 6.3/26.2 ± 6.5
Kogure et al. 2012 [32]	Southeast	Rotterdam	Case–control	20/19	27.8 ± 5.0/27.9 ± 5.2
Maciel et al. 2014 [34]	Southeast	Rotterdam	Cross-sectional	97/–	24.9 ± 5.1/–
Melo et al. 2011^a^ [35]	Southeast	Rotterdam	Cross-sectional	132/146	26.6 ± 5.1/28.9 ± 0.5
Oliveira et al. 2013^b^ [36]	Southeast	Rotterdam	Case–control	42/18	27.4 ± 5.5/31.4 ± 6.1
Pedroso et al. 2012 [37]	Southeast	Rotterdam	Cross-sectional	105/–	29 ± 4.4/–
Pontes et al. 2012 [38]	Southeast	Rotterdam	Cross-sectional	189/–	24.9 ± 5.2/–
Radavelli-Bagatini et al. 2013 [45]	South	Rotterdam	Case–control	80/1500	21.3 ± 0.6/22.7 ± 0.4
Ramos et al. 2015 [46]	South	Rotterdam	Case–control	199/99	22 ± 6/25 ± 7
Santana et al. 2004^c^ [40]	Southeast	NIH	Cohort	21/–	27.2/–
Soares et al. 2016 [27]	Northeast	Rotterdam	Cross-sectional	22/–	26 ± 6.0/–
Tavares et al. 2019 [28]	Northeast	Rotterdam	Cross-sectional	111/–	18–39
Wanderley et al., 2018 [49]	Midwest	Rotterdam	Cross-sectional	83/–	28.79 ± 5.85/–
Wiltgen et al. 2010^d^ [43]	South	Rotterdam	Case–control	195/25	22.3 ± 6.7/29.7 ± 4.29*
Xavier et al. 2018 [42]	Southeast	Rotterdam	Case–control	97/99	30.5 ± 5.1/29.8 ± 7.1
*BMI-matched studies*					
Costa et al., 2008 [25]	Northeast	Rotterdam	Cross-sectional	57/37	25.5 ± 5.3/26.6 ± 5.4
Lauria et al. 2013 [33]	Southeast	Rotterdam	Case–control	40/36	29 (25–34)/30(15–43)
Rocha et al. 2011 [39]	Southeast	Rotterdam	Case–control	142/31	25.1 ± 5.4/27.5 ± 4
Simões et al. 2017 [41]	Southeast	Rotterdam	Case–control	10/10	29.6 ± 1.2/28.6 ± 2.0
Wiltgen et al. 2009 [47]	South	Rotterdam	Case–control	51/44	20.6 ± 5.1/28.9 ± 5.6*

^a^Data are from A phenotype PCOS *vs* controls

^b^Women included in the control group had similar complaints as the ones from the PCOS group, but did not fulfill the diagnostic criteria

^c^Data are from baseline

^d^Data are from A plus B phenotypes PCOS *vs* controls

*p < 0.05 between groups

### Qualitative data synthesis

Table 1 summarizes the characteristics of studies on Brazilian populations. Seven studies were from the Northeast region [23–29], thirteen studies were from the Southeast [30–42], five studies were from the South [43–47], and two studies were from the Midwest region [48, 49]. No data from the North region were available. All the studies used the Rotterdam criteria for diagnosis of PCOS, except by one [40]. In five studies, the control groups and PCOS participants were BMI-matched [25, 33, 39, 41, 47]. Ten out of 27 studies had no control group for comparison [27–30, 34, 37, 38, 40, 48, 49] and thus these studies were not included in the meta-analyses. Another one study was excluded from the meta-analyses because the control group included participants who were hirsute or had irregular menses [36]. Two studies in which data on PCOS participants were presented only as PCOS phenotypes [35, 43] and other two studies that did not present SD values for the variables of interest[29, 34] were equally excluded from the meta-analyses.

In Brazilian women with PCOS, BMI ranged from 24.2 to 31.9 kg/m^2^ (Table 2). In studies without a BMI-matched control group, most PCOS groups had higher BMI than controls [23, 24, 31, 35, 42–46]. The prevalence of obesity in Brazilian women with PCOS diagnosed by Rotterdam criteria was reported in eight studies [26, 28, 34, 39, 42, 46, 48, 49]. Except for one study with non-selected women from primary healthcare services [26] in which the prevalence of obesity was similar to that of the general Brazilian female population [50] in the other seven studies the prevalence of obesity ranged from 31.6 to 56.6 in the Midwest, Southeast, and South, showing quite higher values than those expected for the Brazilian female population (17.9% for women aged 25–34 years) [50] (Table 3).

**Table 2:** Characteristics of Brazilian women with PCOS in the studies included in the systematic review

Name, Year	BMI (PCOS/controls)	Waist circumference (cm) (PCOS /controls)	Blood pressure (mmHg) (PCOS/controls)	Lipid profile (PCOS/controls)	Glucose (mmol/L) (PCOS/controls)	HOMA-IR (PCOS/controls)
*BMI-unmatched studies*						
Avila et al. 2014 [30]	30.6 ± 9.3/–	NA	NA	NA	NA	NA
Azevedo et al. 2011 [23]	29.3 ± 6.7/24.1 ± 4.4*	91.2 ± 15.7/77.1 ± 9.6*	SBP: 114.8 ± 13.3/111.5 ± 10.7* DBP: 72.6 ± 10.7/72.1 ± 10.3	TC (mmol/L): 4.82 ± 1.09/4.57 ± 1.19 HDL (mmol/L): 1.05 ± 0.29/1.39 ± 0.49* LDL (mmol/L): NA TGL (mmol/L): 1.56 ± 0.91/1.13 ± 0.6 *	4.64 ± 0.67/4.25 ± 0.62*	NA
Carvalho et al. 2017 [31]	30.1 ± 5.4/ 23.2 ± 4.23*	98.0 (17.0)/71.5 (16.0)*	NA	NA	4.83 ± 0.4/ 4.7 ± 0.58	3.54(4.8)/1.68(1.6)*
Cerqueira et al. 2010 [24]	27.7 ± 5.4/ 24 ± 4.2*	84.5 ± 11.3/ 78.9 ± 10.0*	SBP: 117.5 ± 11.9/104.0 ± 10.3* DBP: 77.7 ± 9.8/68.7 ± 8.1*	TC (mmol/L): 4.57 ± 0.81/4.11 ± 0.76* HDL (mmol/L): 1.16 ± 0.24/ 1.41 ± 0.43* LDL (mmol/L): 2.29 ± 0.92/2.14 ± 0.75 TGL (mmol/L): 1.3 ± 0.78/0.94 ± 0.43*	4.88 ± 0.68/4.68 ± 0.41*	3.6 ± 3.7/1.9 ± 0.9*
Costa et al. 2012 [29]	29.6 ± 6.6/–	NA	SBP:115.5 ± 13.0/– DBP:73.3 ± 10.3/–	TC (mmol/L): 4.88 ± 1.08/– HDL (mmol/L): 01.14 ± 0.28/– LDL (mmol/L): 3.11 ± 1.07/– TGL (mmol/L): 1.54 ± 0.81/–	4.62 ± 0.68/–	NA
de Medeiros et al. 2014 [48]	29.9 ± 7.0/–	88.0 ± 16.3/–	NA	NA	5.11 ± 0.78/–	1.93 ± 1.21/–
Gabrielli et al. 2012 [26]	24.2 (17.7–30.7)/ 24.1 (18.1–30.1)	74 (56–92)/75 (60–90)	SBP: 119 (99–139)/122 (102–142) DBP: 74 (60–88)/ 74 (59.5–89.5)	NA	NA	NA
Graff et al. 2017 [44]	29.4 ± 6.4/27.2 ± 5.8*	86.6 ± 14.1/83.6 ± 12.3	SBP:118.2 ± 13.0/112.4 ± 11.1* DBP:77.4 ± 9.9/72.8 ± 10.0*	TC (mmol/L): 4.49 ± 0.89/4.5 ± 0.79 HDL (mmol/L): 1.18 ± 0.31/1.31 ± 0.28* LDL (mmol/L): 2.75 ± 0.71/2.72 ± 0.63 TGL (mmol/L): 0.99(0.69–1.53) /0.87(0.6–1.16)	4.85 ± 0.47/4.82 ± 0.44	3.4(1.8–4.7)/2.1(1.5–2.8)*
Kogure et al. 2012 [32]	28.7 ± 4.4/27.1 ± 5.1	NA	NA	TC (mmol/L): 5.42 ± 1.24/5.47 ± 1.19 HDL (mmol/L): 1.38 ± 0.31/1.52 ± 0.28 LDL (mmol/L): 3.29 ± 0.92/3.4 ± 0.96 TGL (mmol/L): 1.65 ± 0.93/1.19 ± 0.75*	5.82 ± 0.99/5.73 ± 1.09	2.3 ± 2.3/1.6 ± 0.8*
Maciel et al. 2014 [34]	29.6 ± 6.9/–	90.1 ± 15.2/–	NA	TC (mmol/L): 4.42 ± 0.82/– HDL (mmol/L): 1.3 ± 0.36/– LDL (mmol/L): 2.55 ± 0.67/– TGL (mmol/L): 1.3 ± 0.7/–	4.95 ± 0.57/–	3.8 ± 3.3/–
Melo et al. 2011^a^ [35]	31.3 ± 8.7/24.4 ± 4.9*	98.6 ± 17.7/84.8 ± 12.4	SBP: 121.5 ± 15.2/111.5 ± 10.8* DBP: 7 8 ± 10.4/71.7 ± 8.2*	TC (mmol/L): 4.76 ± 1.04/4.93 ± 0.84 HDL (mmol/L): 1.16 ± 0.27/1.41 ± 0.28* LDL (mmol/L): 2.9 ± 0.92/2.82 ± 0.72 TGL (mmol/L): 1.49 ± 0.82/0.95 ± 0.44*	5.17 ± 1.41/4.58 ± 0.49*	4.5 ± 4.9/ 1.5 ± 1.1*
Oliveira et al. 2013^b^ [36]	30.2 ± 6.5/27.1 ± 6.2	NA	SBP: 111.8 ± 12.0 / 107.3 ± 15.0 DBP: 70.2 ± 9.0/71 ± 13.5	TC (mmol/L): NA HDL (mmol/L): 1.3 ± 0.38/1.49 ± 0.35 LDL (mmol/L): NA TGL (mmol/L): 1.32 ± 0.91/1.31 ± 0.74	4.82 ± 0.97/4.54 ± 0.47	4.4 ± 6.8 / 2.1 ± 1.1
Pedroso et al. 2012 [37]	31.9 ± 8.2/–	99 ± 16.6)	SBP: 122.5 ± 18.7/– DBP: 79 ± 11/–	TC (mmol/L): 4.86 ± 1.01/– HDL (mmol/L): 1.22 ± 0.32/– LDL (mmol/L): 2.94 ± 0.87/– TGL (mmol/L): 1.48 ± 0.84/–	5.04 ± 1.08/–	NA
Pontes et al. 2012 [38]	31.8 ± 7.6/–	92.2 ± 16.0	SBP: 116.3 ± 14.4/– DBP: 75.1 ± 10.4/–	TC (mmol/L): 4.73 ± 0.9/– HDL (mmol/L): 1.21 ± 0.33/– LDL (mmol/L): 2.90 ± 0.77/– TGL (mmol/L): 1.43 ± 0.91/–	4.87 ± 0.38/–	NA
Radavelli-Bagatini et al. 2013 [45]	31.0 ± 7.9/23.4 ± 4.6*	92.2 ± 18.8/74.5 ± 10.2*	SBP: 124.6 ± 19.9/111.5 ± 13.0* DBP: 79.2 ± 12.5/71.8 ± 10.6*	TC (mmol/L): 4.81 ± 1.16/4.24 ± 0.9* HDL (mmol/L): 1.32 ± 0.28/1.52 ± 0.36* LDL (mmol/L): 2.95 ± 0.98/2.09 ± 0.72* TGL (mmol/L): NA	NA	NA
Ramos et al. 2015 [46]	29.6 ± 6.4/ 27.6 ± 6.0*	89.2 ± 15.0/78.1 ± 11.5*	NA	NA	4.93 ± 0.68/4.91 ± 0.42	NA
Santana et al. 2004^c^ [40]	29.18 ± 7.78/–	89.36 ± 15.23/–	NA	TC (mmol/L): 4.71 ± 0.98/– HDL (mmol/L): 1.01 ± 0.2/– LDL (mmol/L): 3.10 ± 0.86/– TGL (mmol/L): 1.26 ± 0.67/–	4.48 ± 0.59/–	NA
Soares et al. 2016 [27]	29.8 ± 6.1/–	95.4 ± 15.8/–	NA	TC (mmol/L): NA HDL (mmol/L): 1.13 ± 0.35/– LDL (mmol/L): 2.3(1.81–2.69)/– TGL (mmol/L): 1.39(0.71–1.85)/–	4.38 ± 0.53/–	1.9(1.3–3.3)/–
Wanderley et al. 2018 [49]	29.9 ± 5.28/–	92.15 ± 10.72/–	SBP: 123.15 ± 18.38/– DBP: 79.13 ± 11.00/–	TC (mmol/L): 4.73 ± 0.9/– HDL (mmol/L): 1.28 ± 0.33/– LDL (mmol/L): 3.03 ± 0.85/– TGL (mmol/L): 1.25 ± 0.67/–	4.87 ± 0.36/–	NA
Wiltgen et al. 2010^d^ [43]	31 ± 7.98/ 26.97 ± 3.6*	93.79 ± 18.81/ 79.83 ± 8.37*	SBP: 123.1 ± 16.9/115.2 ± 9.5* DBP: 78.9 ± 12.3/73.6 ± 8.3	TC (mmol/L): 4.72 ± 1.13/4.27 ± 0.95 HDL (mmol/L): 1.25 ± 0.29/1.42 ± 0.35 LDL (mmol/L): 2.85 ± 0.96/ 2.47 ± 0.81 TGL (mmol/L): 1.12 (0.76–1.6)/ 0.68 (0.47 – 1.05)*	5.02 ± 1.19/ 4.92 ± 0.45	4.53 (2.6–7.7)/ 2.14 (1.4–3.1)*
Xavier et al. 2018 [42]	28.8 ± 8.1/ 22.9 ± 5.9*	97.0(18.0)/ 82.4(20.0)*	NA	TC (mmol/L): 4.92 ± 0.94/ 4.55 ± 0.83* HDL (mmol/L): 1.19 (0.49) / 1.39 (0.49)* LDL (mmol/L): 2.97 ± 0.82/2.58 ± 0.72* TGL (mmol/L): 1.11 (0.97) /0.93 (0.44)*	6.95 ± 11.2/5.78 ± 7.14	2.8 (1.8)/1.59 (1.2)*
*BMI-matched studies*						
Costa et al. 2008 [25]	27.6 ± 5.8/26.7 ± 4.9	87.8 ± 14.3/83.6 ± 10.1	SBP: 118.9 ± 15.2/113.8 ± 10.9 DBP: 79.9 ± 8.9/73.4 ± 10.2*	TC (mmol/L): 4.37 ± 0.62/4.11 ± 0.83 HDL (mmol/L): 1.23 ± 0.34/ 1.54 ± 0.27* LDL (mmol/L): 2.56 ± 0.65/2.21 ± 0.72* TGL (mmol/L): 1.16 ± 0.55/1.06 ± 0.68		
Lauria et al. 2013 [33]	27.64 ± 5.43/25.99 ± 5.51	91 (83–101)/94 (83–103)	SBP: 120 (110–120)/120 (110–120) DBP: 80 (70–80)/ 80 (70–80)	TC (mmol/L): 0000000000000004.37 (3.67–4.76)/3.67 (3.21–4.34)* HDL (mmol/L): 1.11 (0.88–1.29)/1.01 (0.85–1.14) LDL (mmol/L): 2.77 (2.3–3.26)/2.25 (1.76–2.87)* TGL (mmol/L): 0.91 (0.73–1.22)/0.8 (0.65–1.06)		
Rocha et al. 2011 [39]	29.1 ± 6.17/27.4 ± 6.9	NA	NA	TC (mmol/L): 4.68 ± 0.78/4.28 ± 0.4 HDL (mmol/L): 1.23 ± 0.45/1.51 ± 0.2* LDL (mmol/L): 2.87 ± 0.65/2.63 ± 0.58 TGL (mmol/L): 1.34 ± 0.74/1.32 ± 0.4		
Simões et al. 2017 [41]	28.0 ± 2.4/27.4 ± 2.4	NA	NA	NA		
Wiltgen et al. 2009 [47]	29.5 ± 7.5/29.4 ± 5.4	90.6 ± 16.1/ 85.5 ± 11.6*	NA	TC (mmol/L): 4.85 ± 1.11 / 4.2 ± 0.8* HDL (mmol/L): 1.35 ± 0.27 / 1.32 ± 0.3 LDL (mmol/L) 3.07 ± 0.97/2.46 ± 0.69* TGL (mmol/L): 1.1 (0.77–1.48)/0.73 (0.54–1.21)*		

^a^Data are from A phenotype PCOS *vs* controls

^b^Women included in the control group had similar complaints as the ones from the PCOS group, but did not fulfill the diagnostic criteria

^c^Data are from baseline

^d^Data are from A plus B phenotypes PCOS *vs* controls

* p < 0.05 between the groups. Continuous metabolic variables are not available from Tavares et al. 2019 [24]

**Table 3:** Prevalence of obesity, dyslipidemia, metabolic syndrome, prediabetes and diabetes 2 in Brazilian women with PCOS

Study	N	Region	Obesity (%)	Dyslipidemia (%) ↓ HDL^a^	↑ TGL^b^	MetS (%)	Prediabetes^c^ (%)	Type 2 Diabetes (%)
Avila et al. 2014 [30]	100	Southeast				36		
Costa et al. 2012 [29]	113	Northeast		76.1	33.6	31		
de Medeiroset al. 2014 [48]	288	Midwest	44.3					
Gabrielli et al. 2012 [26]	73	Northeast	13.7					
Maciel et al. 2014 34	97	Southeast	42.3	52.6	22.7	26.8		
Pedroso et al. 2012 [37]^d^	105	Southeast		68.6	5.3	42.9		
Ramos et al. 2015 [46]	199	South		56.6	17.8	24.6	9.7	
Rocha et al. 2011 [28]	142	Southeast	31.6	57.6	28.3			
Tavares et al. 2019 [28]	111	Northeast	44.1	54.1	35.1	33.6	7.2	
Wanderley et al. 2018 [49]	83	Midwest	56.62					
Wiltgen et al. 2010 [43]	195	South	52.5	58.8	22.9	31.3	11.3	3.6
Xavier,et al., 2018 [43]	97	Southeast	42.3					

Rotterdam criteria for all studies

^a^Lower HDL: ≤ 50

^b^Higher TGL: ≥ 150

^c^Prediabetes: IFG and/or IGT

^d^only data of adult women with PCOS were extracted

Table 2 also presents WC, blood pressure, glucose, and lipid profile of Brazilian women with PCOS and control populations. Only three studies [26, 37, 44] did not observe a larger WC in PCOS participants vs. controls. Thirteen studies had no data on WC or a control group for comparison.

Eleven Brazilian studies reported blood pressure data in PCOS and control groups [23–26, 33, 35, 36, 43–46]. Of these studies, eight showed a higher systolic (SBP) or diastolic blood pressure (DBP) in PCOS [23–25, 35, 43–46].

Fasting glucose was measured in 23 studies [23–25, 27, 29, 31–44, 46–49] (Table 2). Glucose levels ranged from 4.38 to 6.95 mmol/L in Brazilian women with PCOS and from 4.25 to 5.78 in controls. In three studies [23, 24, 37] PCOS groups had higher glucose levels than control groups. Eleven studies had no data on glucose levels or a control group for comparison.

Impaired fasting glucose (IFG, fasting glucose between 5.6 and 6.9 mmol/L) [46] and/or impaired glucose tolerance (IGT, glucose levels between 7.8 and 11.1 mmol/L at 120 min after the oGTT) [43] was found in only two studies from the South region (9.7 and 11.3%) and in one from the Northeast (7.2% [28]). Only one study (Table 3) reported prevalence of type 2 diabetes in Brazilian women with PCOS. Out of 195 patients, 3.6% were diabetic.

HOMA-IR, a marker of insulin resistance, was available in 14 studies, and twelve studies also compared HOMA-IR values in PCOS and controls. In ten studies [24, 25, 31, 32, 35, 39, 42–44, 47] HOMA-IR was higher in women with PCOS than in controls (Table 2). Metabolic syndrome was evaluated in Brazilian women with PCOS in the Northeast, Southeast, and South and showed a homogeneous prevalence among the regions, ranging between 24.6 and 42.9% [28–30, 34, 37, 43, 46] (Table 3).

Regarding lipid profile, 19 studies on Brazilian women with PCOS showed TGL levels ranging from 0.91 to 1.65 mmol/L, and in 12 of them values for control groups ranged from 0.68 to 1.32 mmol/L (Table 2). TGL levels were higher in PCOS than controls in seven out of these 12 studies [23, 24, 32, 35, 42, 43, 47]. Twenty-one Brazilian studies assessed HDL cholesterol levels, and 14 of them compared HDL values in PCOS (1.01–1.38 mmol/L) vs. controls (1.01–1.54 mmol/L)[23–25, 32, 33, 35, 36, 39, 42–47] (Table 2). In nine of these studies, HDL was significantly lower in PCOS than in controls [23–25, 35, 39, 42, 44–46]. LDL levels ranged from 2.29 to 3.29 mmol/L in PCOS women from 18 Brazilian studies and from 2.09 to 3.4 mmol/L in controls from 11 studies (Table 2). Five studies [25, 33, 42, 45, 47] reported LDL to be higher in PCOS. Eighteen studies with Brazilian women with PCOS assessed TC levels and 12 compared PCOS with controls, with mean values ranging from 4.37 to 5.42 mmol/L in PCOS and from 4.11 to 5.47 in controls (Table 2). Five studies showed higher TC levels in the PCOS group compared with the control group [24, 33, 42, 45, 47]. Seven studies assessed the prevalence of dyslipidemia in the Northeast, Southeast, and South and showed homogeneous values among the regions, for both lower HDL-cholesterol (ranging between 52.6 and 76.1%) and higher triglycerides (from 5.3 to 35%).

Three studies evaluating referral populations assessed the prevalence of PCOS phenotypes [28, 35, 43]. Phenotypes A + B were more prevalent in these studies (66.4, 81 and 65.8%, respectively).

### Quantitative data synthesis and meta-analysis

Of 27 studies, 12 articles meeting eligibility criteria were included in the meta-analysis [23–25, 31, 32, 39, 41, 42, 44–47], for a total of 995 PCOS and 2,275 control women. All used the Rotterdam criteria to define PCOS. NOS score was 9 in seven studies, 8 in two, 7 in two, and 6 in another one (Table 4).

**Table 4:** Newcastle–Ottawa quality (NOS) assessment scale for studies included in the meta-analysis

Author	Year	Selection	Comparability	Exposure
Azevedo (23)	2011	****	**	***
Carvalho (31)	2017	****	**	***
Cerqueira (24)	2010	**	*	***
Costa (25)	2008	***	*	***
Graff (44)	2017	****	*	***
Kogure (32)	2012	****	**	***
Radavelli − Bagatini (45)	2013	****	*	***
Ramos (46)	2015	****	**	***
Rocha (39)	2011	****	**	***
Simões (41)	2017	****	**	***
Wiltgen (47)	2009	****	**	***
Xavier (42)	2018	***	*	***

Quality of selection (minimum 1–maximum 4 stars); Comparability (minimum 0–maximum 2 stars); Exposure (minimum 1–maximum 3 stars)

#### BMI

**Fig. 2:**
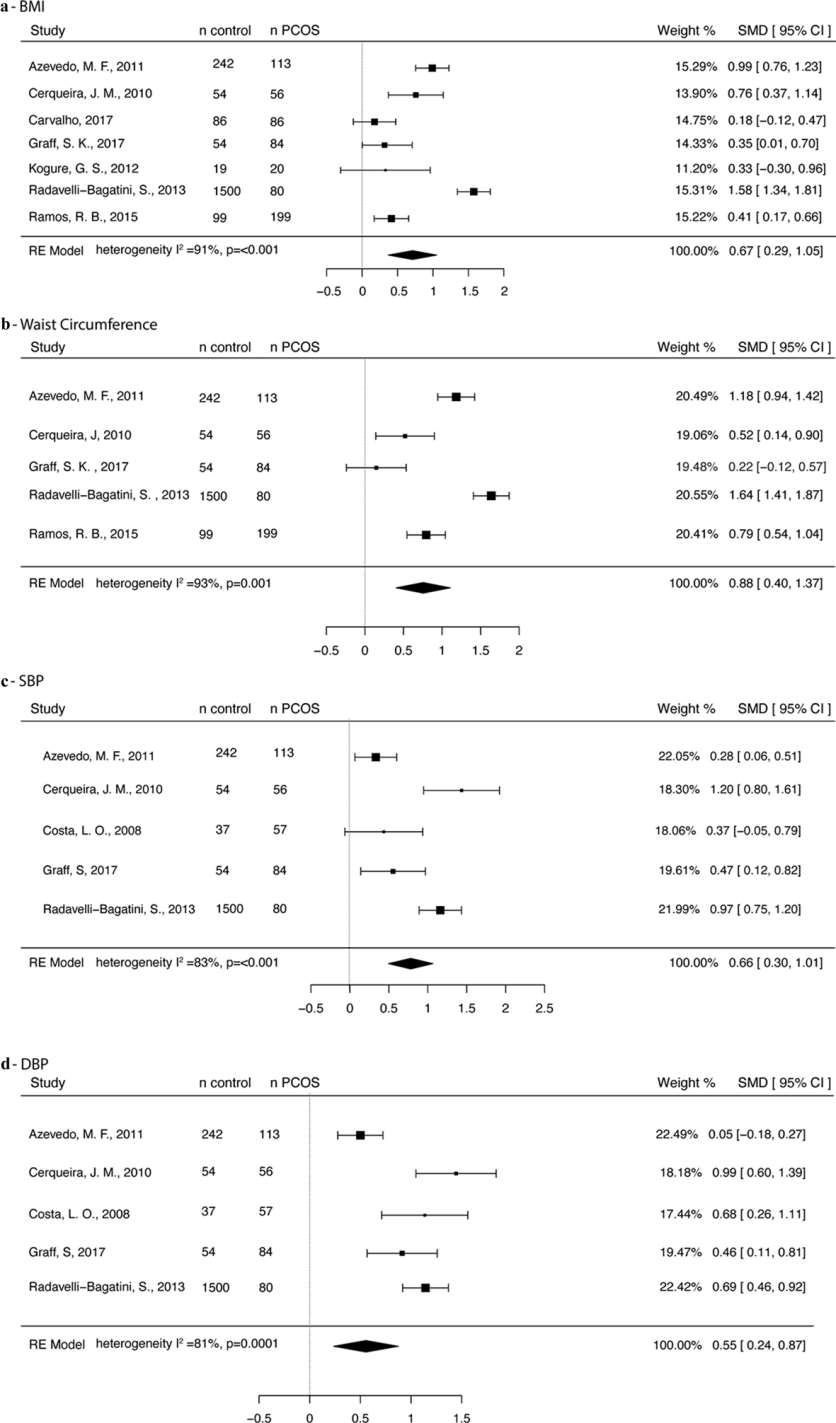
Forest plot showing **a** BMI, **b** waist circumference, **c** systolic blood pressure and **d** diastolic blood pressure

Only BMI-unmatched studies were considered for analysis. Data from seven studies were analyzed [23, 24, 31, 32, 44–46] including 638 PCOS and 2,054 controls. The PCOS group had higher BMI levels than controls (SMD 0.67, 95% CI, 0.29, 1.05). Between-study heterogeneity was high (I^2^ = 91%, p < 0.001) (Fig. 2a).

#### Waist circumference

As for BMI, only BMI-unmatched studies were considered for analysis of waist circumference. Five studies [23, 24, 44–46] with 532 PCOS and 1,949 control participants were considered. The PCOS group had higher waist circumference vs. the control group (SMD 0.88, 95% CI 0.40, 1.37). Between-study heterogeneity was high (I^2^ = 93% p = 0.001) (Fig. 2b).

#### Blood pressure

Five studies entered the meta-analysis of blood pressure [23–25, 44, 45] with 390 PCOS and 1887 control participants. Higher SBP (SMD 0.66, 95% CI 0.30, 1.01) and DBP levels (SMD 0.55, 95% CI 0.24, 0.87) were observed in women with PCOS than in controls (I^2^ = 83%, p < 0.001 for SBP and I^2^ = 81%, p = 0.0001 for DBP) (Fig. 2c and d).

#### Glucose

**Fig. 3:**
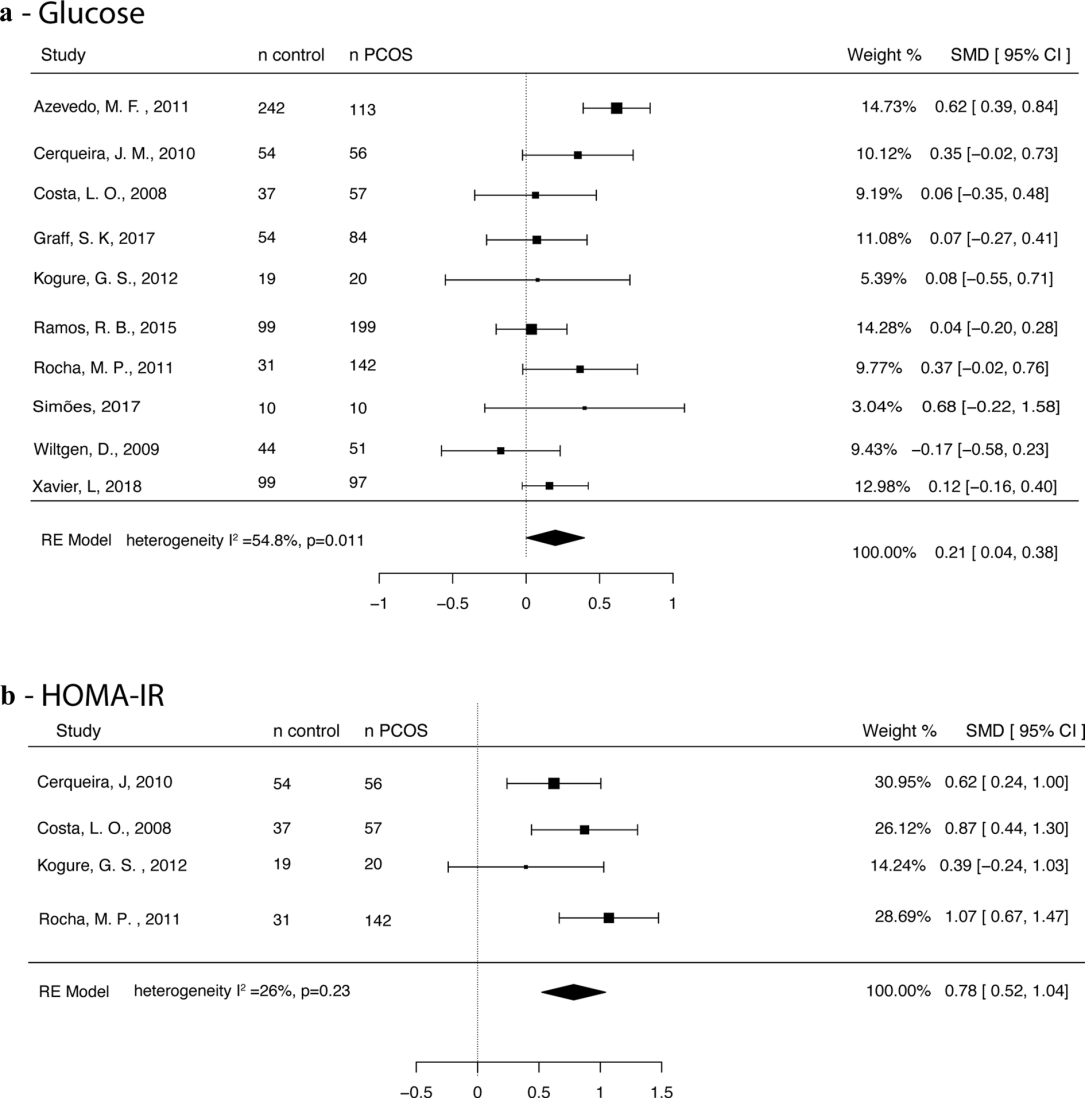
Forest plot showing **a** fasting glucose, **b** HOMA-IR

Ten studies were included for glucose meta-analysis [23–25, 32, 39, 41, 42, 44, 46, 47] including 829 PCOS and 689 control participants. Glucose levels were higher in the PCOS group (SMD 0.21, 95% CI 0.04, 0.38) when compared with controls, with moderate between-study heterogeneity (I^2^ = 54.8%, p = 0.011) (Fig. 3a).

#### HOMA-IR

HOMA-IR was analyzed in four studies [23, 25, 32, 39], for a total of 275 PCOS and 141 controls. HOMA-IR was higher in PCOS vs. controls (SMD 0.78, 95% CI 0.52, 1.04), with low between-study heterogeneity (I^2^ = 26%, p = 0.23) (Fig. 3b).

#### Lipids

**Fig. 4:**
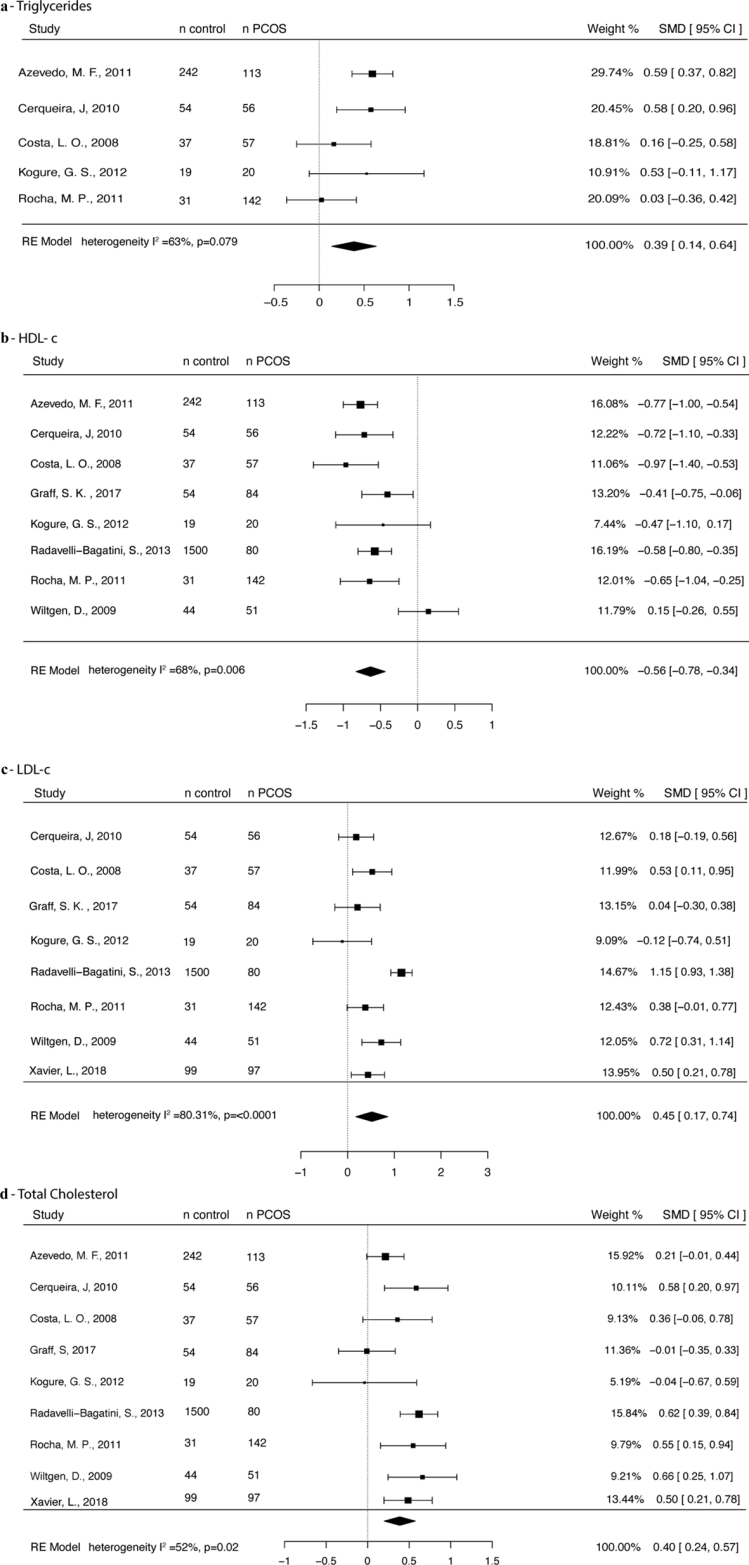
Forest plot showing **a** TGL, **b** HDL-Chol, **c** LDL-Chol, **d** total cholesterol

Data from five studies were available for TGL analysis [23–25, 32, 39], including 388 PCOS and 383 control participants. TGL levels were higher in the PCOS group (SMD of 0.39, 95% CI 0.14, 0.64), with moderate between-study heterogeneity (I^2^ = 63%, p = 0.079) (Fig. 4a). Eight studies were included in the HDL meta-analysis [23–25, 32, 39, 44, 45, 47], with 603 PCOS and 1,981 controls. HDL levels were lower in the PCOS group (SMD − 0.56, 95% CI − 0.78, − 0.34) when compared with controls. Between-study heterogeneity was moderate (I^2^ = 68%, p = 0.006) (Fig. 4b). LDL data were included from eight studies [24, 25, 32, 39, 42, 44, 45, 47] with 587 PCOS and 1,838 controls. LDL levels were higher in PCOS (SMD 0.45 95% CI 0.17, 0.74). Between-study heterogeneity was high (I^2^ = 80.31%, p =  < 0.0001) (Fig. 4c). Nine studies compared total cholesterol levels in PCOS and controls [23–25, 32, 39, 42, 44, 45, 47] for a total of 700 PCOS and 2080 controls. TC levels were higher in PCOS (SMD 0.40 95% CI 0.24, 0.57) than control participants, with moderate between-study heterogeneity (I^2^ = 52%, p = 0.02) (Fig. 4d).

**Fig. 5:**
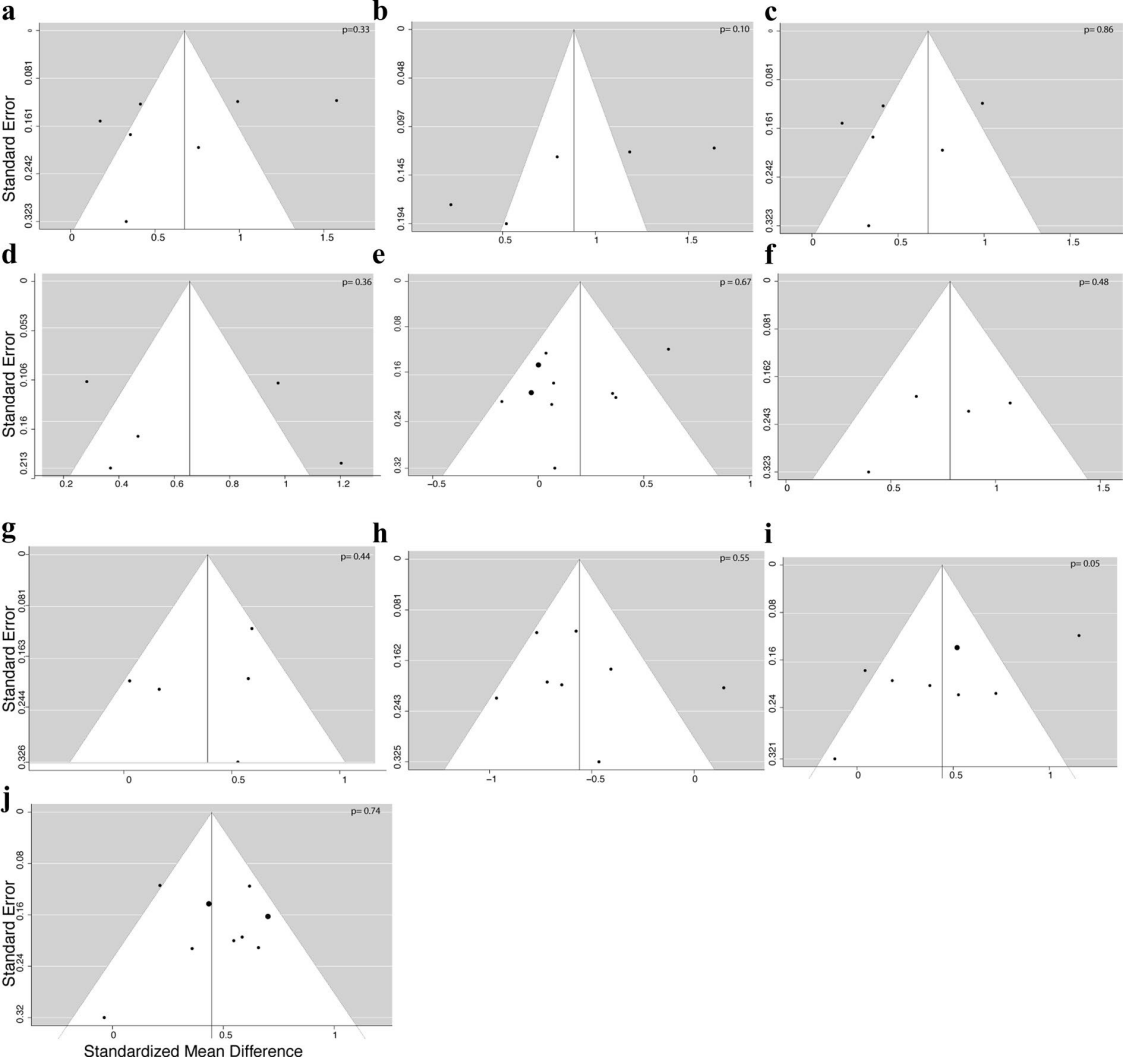
Funnel plots for risk of publication bias for **a** BMI, **b** waist circumference, **c** systolic blood pressure, **d** diastolic blood pressure, **e** glucose, **f** HOMA-IR, **g** TGL, **h** HDL-chol, **i** LDL-chol, **j** total cholesterol

Publication bias may have occurred in comparisons of PCOS vs. controls in LDL analysis (Fig. 5i). Conversely, no publication bias was detected in any other comparisons (p >  = 0.10; Fig. 5a–h and j).

## Discussion

PCOS is a complex condition that affects both the reproductive and the metabolic systems. In this meta-analysis including 12 cross-sectional and case–control studies, for a total of 995 PCOS and 2275 women from Brazil, BMI, waist circumference, blood pressure, glucose, and HOMA-IR were found to be higher in participants with PCOS. Lipid profile was more adverse than that found in non-PCOS women. Even though only observational studies including mostly small samples were examined, the evidence indicates that women with PCOS from different regions of Brazil have a worse cardiometabolic profile than women without PCOS. In addition, the systematic review of 27 observational studies with PCOS women from Brazil indicates that metabolic comorbidities, such as obesity, metabolic syndrome and IFG/IGT are prevalent in Brazilian women with PCOS with no important regional differences. To the best of our knowledge, this is the first systematic review and meta-analysis evaluating metabolic characteristics of women with PCOS in the different regions of Brazil.

Despite the efforts to assess the impact of ethnicity and sociocultural backgrounds on the metabolic traits of PCOS by comparing different populations, very few data are available regarding Latin American countries. In Brazil, the overall ancestry proportion has been described as 0.62 European, 0.21 African and 0.17 Amerindian [51, 52]. However, ancestry proportions seem to differ according to region, as indicated by self-reported skin color rates, in which lower rates of white are found in the North and Northeastern regions (23 and 29% respectively) compared to the South, Southeast and Midwest (78, 55 and 48%, respectively) (sidra.ibge.gov.br/Tabela/3175 – accessed on 06/24/2020). Although these genetic backgrounds could impact the phenotypic heterogeneity of PCOS, the evidence emerging from the present study rather suggests similarities in metabolic traits throughout the different regions of the country, a finding that could potentially inform public health care systems, preventive programs, and policies targeting women with PCOS in Brazil.

In the present systematic review and meta-analysis, women with PCOS from Brazil had higher BMI and worse metabolic status. Analysis of the available data showed that in women with PCOS, obesity, metabolic syndrome, and IFG/IGT were more frequent than in Brazilian women from the general population of same age [50]. Of note, most of these studies were from referral populations, which may have influenced the prevalence of comorbidities at least to a certain extent [53].

While the current information on the frequency of obesity in women with PCOS from different countries comes from only a few studies, generally with a small number of participants, or from studies reporting obesity as a secondary outcome, the present data could indicate that the prevalence of obesity in Brazilian women with PCOS, varying from 31.6 to 56.6%, may be close to that of Scandinavian countries (42% [54]; 35% [55]), and half way between that observed in U.S. women with PCOS diagnosed with Rotterdam criteria (65%) [11, 15] and that found in Mediterranean countries (8 and 31%) [6, 7, 11]. The prevalence of metabolic syndrome (24.6–42.9% in Brazilian women with PCOS) seems to be similar to that found in the U.S. (34.6% [15]; 43% [13]), and much higher than that observed in Mediterranean countries (10% [6]; 6.6% [7]).

The data regarding type 2 diabetes were too few for analysis, with only one study reporting a prevalence of diabetes of 3.6% in 195 Brazilian women with PCOS. Thus, further studies are needed in order to determine the frequency of diabetes in women with PCOS across the country [56]. In turn, the prevalence of impaired fasting glucose in PCOS women from the South and Northeast of Brazil was similar to that of Mediterranean countries [7] and lower than that observed in the U.S. [12–14].

Taken together, these data suggest that PCOS-related metabolic alterations are less prevalent in Brazil than in the U.S., where the mean body weight of the general population is higher than in most other countries [57]. Also, additional studies are warranted, covering other underrepresented regions, such as the North of Brazil.

Another metabolic feature of PCOS is insulin resistance, with higher risk of impaired glucose tolerance, type 2 diabetes, and gestational diabetes [58–60]. A recent study showed a pooled PCOS prevalence of 24% (95% CI 15; 34) in adolescent and adult women with type 1 diabetes, which is markedly higher than the general population [61]. In our systematic review and meta-analysis, we found Brazilian women with PCOS presented increased HOMA-IR. Previous studies have found that women with PCOS, as opposed to controls, have significantly decreased insulin sensitivity with increasing BMI [58, 62] and low-grade chronic inflammation [63, 64]. Previous meta-analyses including different populations have shown higher odds for MetS among women with PCOS [65, 66]. However, in both studies very few data from Latin American populations were available. In turn, due to the paucity of available data, additional studies assessing prediabetes and diabetes and distinct PCOS phenotypes in different countries of Latin America are warranted and could produce relevant information for the primary and secondary prevention of these PCOS-related metabolic comorbidities in the region.

Considering the dearth of information, the present systematic review and meta-analysis provides a comprehensive overview of metabolic and anthropometric variables among women diagnosed with PCOS in Brazil. A major strength of our study is the extensive search strategy, covering the main databases to avoid missing any relevant information, with active search for studies published also in Portuguese language. Limitations are the small number of studies in view of the size of the region, the small sample sizes, and the possible sources of heterogeneity across the studies. However, there are no other similar analyses in the literature. Thus, this study represents the first evidence to characterize the metabolic profile of women with PCOS in the context of ethnicities and sociocultural backgrounds in Brazil.

## Conclusions

The present results indicate that women with PCOS from different regions of Brazil have worse anthropometric and metabolic profiles than women from the same regions without PCOS. The prevalence of metabolic changes is intermediate in Brazil in comparison with other countries. Regarding the prevalence of diabetes, the evidence produced is not conclusive, suggesting that additional studies are warranted and could produce invaluable results in the context of PCOS.

## Supplementary Information

**Additional file 1.** Medical subject headings (MeSH) used in the search.

## Data Availability

All data generated or analyzed during this study are included within the article and its supplementary information file.

## Abbreviations

BMI: Body mass index
DBP: Diastolic blood pressure
DM: Diabetes mellitus
HDL: High-density lipoprotein
HOMA: Homeostatic Model Assessment
IFG: Impaired fasting glucose
IGT: Impaired glucose tolerance
IR: Insulin resistance
LDL: Low-density lipoprotein
MetS: Metabolic syndrome
NOS: Newcastle–Ottawa scale
PCOS: Polycystic ovary syndrome
SBP: Systolic blood pressure
SMD: Standardized mean difference
TC: Total cholesterol
TGL: Triglycerides
WC: Waist circumference
