# Supplementary material for: Metabolic profile of women with PCOS in Brazil: a systematic review and meta-analysis
Source: Diabetol Metab Syndr. 2021 Feb 16;13:18. doi: 10.1186/s13098-021-00636-5 (PMC7885437; doi:10.1186/s13098-021-00636-5)
Supplement: Supplementary file 1 — Additional file 1. Medical subject headings (MeSH) used in the search. [file 13098_2021_636_MOESM1_ESM.docx]

**Additional file 1**

**Medical subject headings (MeSH) used in the search**

The following medical subject headings (MeSH) were used in the search:

“Polycystic Ovary Syndrome” [MeSH] OR “Ovary Syndrome, Polycystic” OR “Syndrome, Polycystic Ovary” OR “PCOS” OR “Polycystic Ovarian Syndrome” OR “Ovarian Syndrome, Polycystic” AND “Body Mass Index” [MeSH] OR “Metabolic Syndrome” OR “Glucose Intolerance” [MeSH] OR “Intolerance, Glucose” OR “Intolerances, Glucose” OR “Diabetes Mellitus, Type 2” [MeSH].
